# Development and validation of a concussion risk prediction model using 2023 National Health Interview Survey (NHIS) data

**DOI:** 10.1097/MD.0000000000047935

**Published:** 2026-03-06

**Authors:** Senyuan Yang, Yashi Chen, Shunqiu Huang, Yeling Deng, Xiaobin Zhou, Yong Li

**Affiliations:** a Department of Neurosurgery, The First Affiliated Hospital of Shantou University Medical College, Shantou, Guangdong, China.

**Keywords:** concussion, exploratory model, mild traumatic brain injury, NHIS database, nomogram

## Abstract

Concussions are complex, as patients often present with nonspecific symptoms, requiring timely evaluation and accurate diagnosis. This study, using the National Health Interview Survey database, aimed to explore and validate a concussion risk model to support diagnostic decision-making and patient treatment supervision. This study included demographic and clinical data of 14,275 subjects in 2023. Predictive indicators were selected using least baseline characteristics and least absolute shrinkage and selection operator regression analysis, and a risk nomogram model was constructed. The model was evaluated using calibration curves, the area under curve of receiver operating characteristic, and decision curve analysis. The eligible concussion group (n = 363) and the nonconcussion group (n = 13,912) from the National Health Interview Survey database exhibited significant differences in 9 baseline characteristics (*P* <.05). Age, education level, general health, family income-to-poverty ratio, marital status, mental health, anxiety, behavior, and industry were found to be predictive indicators for patients with concussion. The model built using these predictive indicators demonstrated an area under curve of 0.712 in the receiver operating characteristic curve (95% CI: 0.68647 − 0.73671), indicating good predictive performance. The nomogram showed a strong association between the predicted and actual risks, with high calibration. Decision curve analysis confirmed strong discriminative ability of the model. The exploratory model based on 9 predictive indicators served as a valuable decision-making tool for clinicians. In concussion patients, these predictive indicators could be closely monitored in clinical practice, allowing for timely intervention to improve prognosis.

## 1. Introduction

Traumatic brain injury (TBI) is a significant public health issue that impacts the lives of individuals globally. It is estimated that between 64 and 74 million people suffer from TBI annually, with nearly half of the global population expected to experience at least 1 TBI during their lifetime.^[1,2]^ Concussion, a form of TBI caused by a complex physiological and pathological process, is defined as a transient disturbance in brain function due to trauma and is often referred to as a mild TBI.^[3]^ In clinical practice, concussions are frequently underdiagnosed or overlooked due to their often atypical symptoms and the absence of obvious intracranial injuries detected by brain imaging, such as computed tomography scans or magnetic resonance imaging. While most individuals recover from concussion within 2 to 4 weeks, a significant minority experience persistent postconcussive symptoms (PPCS), which encompass a series of physical, cognitive, and emotional symptoms or sequelae associated with concussion.^[4–6]^ Furthermore, untreated or recurrent concussions may develop into progressive neurodegenerative diseases, ultimately resulting in irreversible neurological damage.^[1,7–9]^ The occurrence of recurrent concussion exhibits notable population specificity, with certain groups demonstrating particularly elevated susceptibility. Athletes participating in contact sports represent a well-recognized high-risk population. A surveillance study reported that during the 2011–2012 to 2014–2015 academic years, a total of 1410 student-athletes sustained 1485 concussions across 13 sports, including baseball, basketball, football, and ice hockey, encompassing 1094 team seasons.^[10]^ In addition, a national investigation from the United States revealed that adolescents engaged in organized sports such as baseball, basketball, gymnastics, ice hockey, lacrosse, soccer, track and field, and weightlifting had significantly higher odds of reporting multiple diagnosed concussions compared with their nonparticipating counterparts.^[11]^ Military personnel also face an elevated risk due to repeated exposure to blast overpressure from firearms and explosive devices,^[12]^ which has been shown to correlate with both structural brain alterations and functional impairments.^[13]^ Furthermore, older adults constitute another vulnerable group for TBI, largely attributable to falls, and demonstrate a substantially higher risk of postconcussive cognitive impairment than other age groups.^[14]^ Collectively, these high-risk populations face not only immediate neurological consequences but also potential long-term sequelae, highlighting the critical importance of early risk identification and preventive strategies. Previous studies have made significant efforts to explore the pathophysiological mechanisms of concussion and identify effective treatments; however, the exact mechanisms of concussion remain poorly understood, and no consensus on treatment has been reached.^[4,15]^ As a result, early recognition and intervention are critical.

Recently, some researchers have attempted to address the factors associated with concussion by presenting various predictive models for clinical use. However, these studies have been largely limited to athletes with sports-related injuries rather than the general population.^[16,17]^ Another study using population-based data from the National Health Interview Survey (NHIS) which is a cross-sectional survey of U.S. adults aged 18 years or older who are not institutionalized, illustrated the relationship between prior concussions and current mental and social well-being.^[18]^ However, these studies have primarily focused on pediatric populations. In the field of adult concussion, risk prediction models based on large-scale general population data that integrate multidimensional factors (e.g., sociodemographic characteristics and health status) are still lacking. Consequently, reliable tools for the early identification of concussion in adults are not yet available, and this research gap limits the clinical guidance value for the prevention and control of adult concussion.

In this study, we investigated key predictive indicators of concussion in adults using data from the 2023 NHIS. Our analysis focused solely on the 2023 dataset. We developed a concussion risk prediction model integrating 4 data types – social demographics, health status, comorbidities, and lifestyle factors – using machine learning algorithms. In future research, we plan to use external datasets to further validate the model’s generalizability and stability. This model aims to deepen understanding of concussion’s physiological and pathological mechanisms in adults, guide future research, and support clinical decision-making and patient management.

## 2. Materials and methods

### 2.1. Study population and data collection

The NHIS database (https://www.cdc.gov/nchs/nhis/) is an annual national survey conducted by the National Center for Health Statistics of the Centers for Disease Control and Prevention (CDC). Its aim is to offer a typical sample of the civilian, non-non populace of the United States. The NHIS procedure was examined and sanctioned by the NCHS Institutional Review Board, and written informed consent was acquired from all the participants. The NHIS employs a complicated, stratified, multi-phase sampling plan to choose households from random clusters. In these households, a random selection of adults aged 18 and above is made to take part in a comprehensive survey encompassing health conditions, health services, lifestyle risk elements, common diseases, and other health-related issues. Information is gathered via face-to-face interviews carried out by trained investigators.

**Figure 1.**
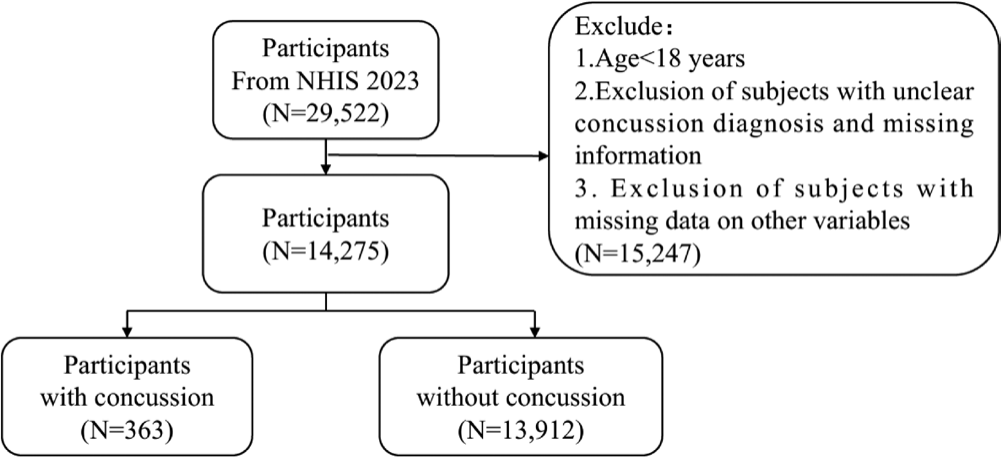
Flow diagram of selection process.

The 2023 NHIS is the latest publicly available database, with earlier versions differing in the collection of concussion-related variables. Therefore, this study only included adults (aged ≥18 years) from the 2023 NHIS data who self-reported a history of concussion. Participants were selected based on self-reported concussion status, with exclusion criteria including: individuals aged <18 years; those with incomplete or ambiguous responses to concussion-related questions; and those with missing data on other variables. Specific classification criteria: Sample classification criteria: 5 variables related to concussion (TBILCDCMG_A, TBIHLSBMC_A, TBISPORT_A, TBILEAGUE_A, TBIEVAL_A, etc) were retained to define the outcome variable If the value of any variable related to concussion is 1, it is marked as concussion (concussion group) If all the variables of concussion are equal to 2 and there is no 1 mean the group is labeled control (control group) If all are empty or other values, they are marked as NA and removed The initial data for concussion cases were 938, control was 27,601, and NA was 983, Excluding age, Hispanic identity, body mass index (BMI) category, health status, mental health treatment, Medicare coverage, history of anxiety, mental health medication use, industry type, occupational type unclassified/missing/unknown, Regroup by age: 18 to 24 = 1, 25 to 44 = 2, 45-63 = 3, and ≥64 = 4 Gender is retained only as 1 (male) and 2 (female) Educational level (EDUCP_A): Middle school is 2; university and above is 3 Race (RATCAT_A): White = 1; Black = 2; Other race = 3 After screening, there were 363 remaining cases of concussion and 13,912 controls (Fig. 1).

### 2.2. Diagnosis of concussion

The study participants were selected from the NHIS database, specifically using data from 2023. The focus was on the “Sample Adult Interview.” To identify individuals who had experienced a concussion, the questionnaire TBILCDCMG_A was used. The TBIHLSBMC_A was employed to assess the presence of concussion-related symptoms. To examine the context surrounding the concussion, TBISPORT_A was used, while TBILEAGUE_A was utilized to identify individuals who experienced a concussion during organized sports participation or practice. Finally, TBIEVAL_A was employed to assess whether the individual had received a concussion or brain injury evaluation. The specific contents of these 5 questionnaires were presented in Table S1, Supplementary Digital Content, https://links.lww.com/MD/R495. Individuals who answered affirmatively were classified as concussion patients.

### 2.3. Covariates

In this investigation, a range of covariates were included to account for their potential influence on concussion. These variables were categorized into demographic characteristics, health status, comorbidities, lifestyle factors, and occupational data: demographic characteristics: age (18–29, 30–44, 45–64, and ≥ 65 years), race, gender (female or male), education level (high school or less, some college, and ≥ 4 years of college education), marital status (either married/cohabitating or divorced/separated/widowed/never married), family income-to-poverty ratio (≥65,000 USD, 35,000–64,999 USD, and <35,000 USD). health status data: BMI (underweight, healthy weight, overweight, obesity), general health status, mental health (participants were classified based on whether they had received counseling or treatment from a mental health practitioner in the past 12 months), health insurance (yes/no). Comorbid conditions: anxiety (yes/no). Lifestyle data: behavior (participants were asked whether they had used medication for emotional, attention, behavior, or mental health issues in the past 12 months). Industry and occupation: industry (participants were classified into 79 different industries), occupation (participants were classified into 23 different occupational categories). These covariates were included to control for potential confounding effects when constructing a risk prediction model associated with concussion. The covariates and their corresponding identification numbers were presented in Table S2, Supplementary Digital Content, https://links.lww.com/MD/R495.

### 2.4. Baseline characteristics and identification of predictive indicators

To investigate the relationship between different baseline characteristics and concussion, patients were separated into the concussion and nonconcussion groups. The differences in various baseline data between the 2 groups of subjects were analyzed using *t*-tests or chi-square tests (*P* <.05), performed by the table 1 package (v 0.13.2).^[19]^

To identify the predictive indicators affecting concussion patients and build a risk prediction model, the glmnet package (v 4.1.4)^[20]^ was utilized to perform least absolute shrinkage and selection operator (LASSO) analysis on the characteristics that showed differences between the concussion and nonconcussion groups. In order to prevent overfitting, the data set categories were weighted and cross validated by 3 folds. LASSO regression achieves variable exclusion through L1 regularization, and its core mechanism is to shrink the coefficients of unimportant variables to zero via the penalty term.

### 2.5. Establishment and validation of nomogram

In order to prevent overfitting, the data set was weighted by category and cross validated by 3 folds. To appraise the predictive power of the predictive indicators, the rms package (v 6.5.1)^[21]^ was utilized to construct a nomogram. Points were assigned to each indicator based on their impact on concussion as reflected in the nomogram model. The total of these points yielded total points, which was then employed to forecast the survival probabilities of concussion patients. Next, the ResourceSelection package (v 0.3.5) (https://github.com/psolymos/ResourceSelection/issues) was employed to generate calibration curves to assess the predictive ability of the nomogram model. The Hosmer–Lemeshow (H–L) test was employed as a model fit indicator; a *P*-value exceeding .05 implies that there is no notable disparity between the predicted and observed values. A mean absolute error of <0.1 indicates that the discrepancy between the actual and predicted risk is very small. Additionally, to evaluate the diagnostic worth of the nomogram, receiver operating characteristic (ROC) curves (with an area under the curve (AUC) value >0.7 considered indicative of good predictive ability) and decision curve analysis (DCA) were conducted using rmda (v 1.6)^[22]^ and pROC (v 1.18.0)^[23]^ packages, respectively.

### 2.6. Statistical analysis

Categorical data were expressed as numbers (percentages). A *P*-value of <.05 signified that analyses were statistically significant. The entire analysis was executed using R software (v 4.2.2). The statistical methods, including LASSO regression and nomogram validation, were reviewed and approved by a qualified statistician to ensure their appropriateness and validity for this study.

## 3. Results

### 3.1. Characteristics of the baseline population

Table 1 displays a complete comparison of demographic and variable data between the concussion and nonconcussion groups. It was noteworthy that there were considerable differences in the distribution of age, family income-to-poverty ratio, education level, general health status, marital status, mental health, anxiety, behavior, and industry between the concussion and nonconcussion groups (*P* <.05).

**Table 1:** Demographic and variable data comparison between concussion and nonconcussion groups.

	Level	Concussion	Nonconcussion	*P*-value
n	–	363	13,912	–
Age (%)	18–24	49 (13.499)	1082 (7.777)	**<.0001**
	25–44	167 (46.006)	5774 (41.504)	
	45–64	94 (25.895)	5059 (36.364)	
	≥65	53 (14.601)	1997 (14.355)	
Gender (%)	Male	187 (51.515)	7449 (53.544)	.4767
	Female	176 (48.485)	6463 (46.456)	
Race (%)	Hispanic (Mexican/Mexican American)	33 (9.091)	1427 (10.257)	.5214
	Hispanic (all other groups)	25 (6.887)	1120 (8.051)	
	Non-Hispanic	305 (84.022)	11,365 (81.692)	
Education level (%)	High school or below	12 (3.306)	881 (6.333)	**.0484**
	High school	81 (22.314)	3225 (23.181)	
	High school or above	270 (74.380)	9806 (70.486)	
Marital Status (%)	Married	133 (36.639)	6708 (48.217)	**<.0001**
	Unmarried cohabitating	31 (8.540)	1212 (8.712)	
	Neither	199 (54.821)	5992 (43.071)	
Family Income-to-Poverty Ratio (%)	<35,000 USD	36 (9.917)	877 (6.304)	**.0046**
	35,000–64,999 USD	61 (16.804)	1965 (14.124)	
	≥65,000 USD	266 (73.278)	11,070 (79.572)	
BMI (%)	Underweight	7 (1.928)	165 (1.186)	0.4204
	Healthy weight	117 (32.231)	4205 (30.226)	
	Overweight	119 (32.782)	4937 (35.487)	
	Obesity	120 (33.058)	4605 (33.101)	
General Health Status (%)	Excellent	75 (20.661)	3759 (27.020)	**<.0001**
	Very good	128 (35.262)	5298 (38.082)	
	Good	100 (27.548)	3745 (26.919)	
	Fair	54 (14.876)	1000 (7.188)	
	Poor	6 (1.653)	110 (0.791)	
Mental health (%)	Yes	65 (17.906)	1234 (8.870)	**<.0001**
	No	298 (82.094)	12,678 (91.130)	
Health Insurance (%)	Not purchased	39 (10.744)	1313 (9.438)	.4544
	Purchased	324 (89.256)	12,599 (90.562)	
Anxiety (%)	Yes	74 (20.386)	1105 (7.943)	**<.0001**
	No	289 (79.614)	12,807 (92.057)	
Behavior (%)	Yes	22 (6.061)	379 (2.724)	**.0003**
	No	341 (93.939)	13,533 (97.276)	
Industry (%)	Crop production	2 (0.551)	124 (0.891)	**.0041**
	Animal production and aquaculture	3 (0.826)	62 (0.446)	
	Forestry and logging	0 (0.000)	13 (0.093)	
	Fishing, hunting, and trapping	0 (0.000)	8 (0.058)	
	Support activities for agriculture and forestry	0 (0.000)	18 (0.129)	
	Oil and gas extraction	0 (0.000)	7 (0.050)	
	Mining (except oil and gas)	0 (0.000)	17 (0.122)	
	Support activities for mining	0 (0.000)	45 (0.323)	
	Utilities	1 (0.275)	136 (0.978)	
	Construction	27 (7.438)	1055 (7.583)	
	Food manufacturing	3 (0.826)	149 (1.071)	
	Beverage and tobacco product manufacturing	3 (0.826)	40 (0.288)	
	Textile mills	0 (0.000)	10 (0.072)	
	Textile product mills	2 (0.551)	11 (0.079)	
	Apparel manufacturing	0 (0.000)	16 (0.115)	
	Leather and allied product manufacturing	0 (0.000)	1 (0.007)	
	Wood products manufacturing	1 (0.275)	32 (0.230)	
	Paper manufacturing	0 (0.000)	26 (0.187)	
	Printing and related support activities	0 (0.000)	29 (0.208)	
	Petroleum and coal products manufacturing	0 (0.000)	23 (0.165)	
	Chemical manufacturing	1 (0.275)	130 (0.934)	
	Plastics and rubber products manufacturing	0 (0.000)	35 (0.252)	
	Nonmetallic mineral product manufacturing	2 (0.551)	38 (0.273)	
	Primary metal manufacturing	0 (0.000)	37 (0.266)	
	Fabricated metal product manufacturing	2 (0.551)	89 (0.640)	
	Machinery manufacturing	0 (0.000)	127 (0.913)	
	Computer and electronic product manufacturing	1 (0.275)	89 (0.640)	
	Electrical equipment, appliances, and components manufacturing	2 (0.551)	24 (0.173)	
	Transportation equipment manufacturing	4 (1.102)	219 (1.574)	
	Furniture and related products manufacturing	0 (0.000)	34 (0.244)	
	Miscellaneous manufacturing	5 (1.377)	150 (1.078)	
	Wholesale trade, durable goods	2 (0.551)	119 (0.855)	
	Wholesale trade, nondurable goods	4 (1.102)	145 (1.042)	
	Nondesignated wholesale trade	0 (0.000)	7 (0.050)	
	Motor vehicle and parts dealers	6 (1.653)	128 (0.920)	
	Furniture and home furnishings stores	2 (0.551)	26 (0.187)	
	Electronics and appliance stores	0 (0.000)	30 (0.216)	
	Building material and garden equipment and supplies dealers	1 (0.275)	116 (0.834)	
	Food and beverage stores	6 (1.653)	226 (1.624)	
	Health and personal care stores	0 (0.000)	94 (0.676)	
	Gas stations	2 (0.551)	31 (0.223)	
	Clothing, footwear, jewelry, luggage, and leather goods stores	5 (1.377)	90 (0.647)	
	Sporting goods, hobby, book, and music stores	3 (0.826)	38 (0.273)	
	Department stores	6 (1.653)	206 (1.481)	
	Miscellaneous store retailers	3 (0.826)	89 (0.640)	
	Non-store retailers and non-designated retail trade	4 (1.102)	101 (0.726)	
	Transportation (including transportation support activities)	12 (3.306)	450 (3.235)	
	Postal services, couriers, and messengers	5 (1.377)	201 (1.445)	
	Warehousing and storage	1 (0.275)	91 (0.654)	
	Newspaper, periodical, book, and software publishing	1 (0.275)	59 (0.424)	
	Motion picture and sound recording industries	5 (1.377)	42 (0.302)	
	Broadcasting and telecommunications	3 (0.826)	82 (0.589)	
	Libraries and archives, internet publishing, web search portals, data processing and hosting services, and other information services	1 (0.275)	77 (0.553)	
	Monetary authorities-central bank	3 (0.826)	192 (1.380)	
	Credit intermediaries and related activities	1 (0.275)	149 (1.071)	
	Securities, commodity contracts, and other financial investments and related activities	1 (0.275)	116 (0.834)	
	Insurance carriers and related activities	10 (2.755)	274 (1.970)	
	Real estate	5 (1.377)	247 (1.775)	
	Automotive and other consumer goods rental services	2 (0.551)	21 (0.151)	
	Commercial, industrial, and other intangible asset services (excluding copyrighted works)	0 (0.000)	13 (0.093)	
	Professional, scientific, and technical services	32 (8.815)	1352 (9.718)	
	Management of companies and enterprises	0 (0.000)	11 (0.079)	
	Administrative and support and waste management and remediation services	14 (3.857)	605 (4.349)	
	Educational services	32 (8.815)	1262 (9.071)	
	Ambulatory health care services	22 (6.061)	778 (5.592)	
	Hospitals	12 (3.306)	646 (4.643)	
	Nursing and residential care facilities	3 (0.826)	199 (1.430)	
	Social assistance	9 (2.479)	288 (2.070)	
	Performing arts, spectator sports, and related industries	7 (1.928)	97 (0.697)	
	Museums, historical sites, and similar institutions	4 (1.102)	39 (0.280)	
	Amusement, gambling, and recreation industries	5 (1.377)	176 (1.265)	
	Accommodations	10 (2.755)	123 (0.884)	
	Food services and drinking places	18 (4.959)	652 (4.687)	
	Repair and maintenance	9 (2.479)	172 (1.236)	
	Personal services (e.g., barbershops, beauty salons, nail salons, laundromats, funeral homes, and cemeteries)	8 (2.204)	208 (1.495)	
	Religious organizations, grantmaking organizations, civic organizations, labor organizations, professional organizations, and similar organizations	5 (1.377)	206 (1.481)	
	Private households	2 (0.551)	68 (0.489)	
	Public administration	18 (4.959)	773 (5.556)	
	Armed forces	0 (0.000)	3 (0.022)	
Occupation (%)	Management occupations	34 (9.366)	1651 (11.867)	.0505
	Business and financial operations occupations	16 (4.408)	995 (7.152)	
	Computer and mathematical occupations	16 (4.408)	681 (4.895)	
	Architecture and engineering occupations	6 (1.653)	354 (2.545)	
	Life, physical, and social science occupations	5 (1.377)	189 (1.359)	
	Community and social service occupations	6 (1.653)	254 (1.826)	
	Legal occupations	5 (1.377)	173 (1.244)	
	Education, training, and library occupations	27 (7.438)	854 (6.139)	
	Arts, design, entertainment, sports, and media occupations	9 (2.479)	294 (2.113)	
	Healthcare practitioners and technical occupations	21 (5.785)	857 (6.160)	
	Healthcare support occupations	14 (3.857)	418 (3.005)	
	Protective service occupations	13 (3.581)	269 (1.934)	
	Food preparation and serving-related occupations	19 (5.234)	632 (4.543)	
	Building and grounds cleaning and maintenance occupations	17 (4.683)	487 (3.501)	
	Personal care and service occupations	18 (4.959)	318 (2.286)	
	Sales and related occupations	20 (5.510)	1028 (7.389)	
	Office and administrative support occupations	35 (9.642)	1496 (10.753)	
	Farming, fishing, and forestry occupations	1 (0.275)	85 (0.611)	
	Construction and extraction occupations	19 (5.234)	737 (5.298)	
	Installation, maintenance, and repair occupations	17 (4.683)	427 (3.069)	
	Production occupations	15 (4.132)	724 (5.204)	
	Transportation and material moving occupations	30 (8.264)	984 (7.073)	
	Military-specific occupations	0 (0.000)	5 (0.036)	

Table 1 presents a comprehensive comparison of demographic and health-related characteristics between individuals with concussion and those without concussion. The data includes various factors such as age, gender, race, education level, marital status, family income, BMI, general health status, mental health, health insurance, anxiety, behavior, industry, and occupation. The *P*-value is provided to indicate the statistical significance of the differences observed between the 2 groups. A lower *P*-value (typically <.05) suggests a significant difference between the concussion and nonconcussion groups for the respective variable. Bold values indicate statistical significance (*P* < .05).

BMI = body mass index.

Among individuals without a concussion, a higher proportion had education levels of “High school or below” (6.3% vs 3.3%) and “High school” (23.2% vs 22.3%). However, a greater proportion of concussion patients had received more education (74.4% vs 70.5%; *P* = .0484). Regarding marital status, a larger proportion of individuals without a concussion were married (48.2% vs 36.6%) or unmarried cohabitating (8.7% vs 8.5%). In contrast, more concussion patients were divorced, separated, widowed, or never married (54.8% vs 43.1%; *P* <.0001). For the family income-to-poverty ratio, a higher percentage of concussion patients had incomes of <35,000 USD (9.9% vs 6.3%) and 35,000–64,999 USD (16.8% vs 14.1%), while more individuals without a concussion had incomes of ≥ 65,000 USD (79.6% vs 73.3%; *P* = .0046). In terms of general health status (*P* <.0001), anxiety (*P* <.0001), and behavior (*P* = .0003), a greater proportion of concussion patients reported abnormal physical and mental states. Moreover, individuals working in the performing arts, spectator sports, and related industries were more likely to experience concussions.

### 3.2. Selected variables based on LASSO

**Figure 2.**
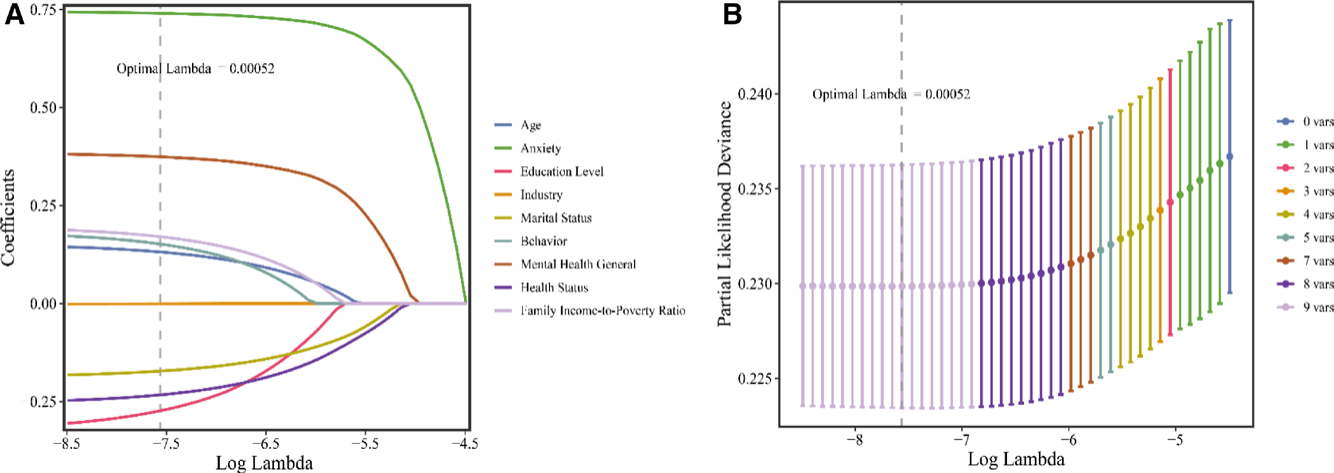
(A) The coefficient distribution of the LASSO regression model; (B) the cross-validation error plot of the LASSO regression model. LASSO = least absolute shrinkage and selection operator.

The aforementioned baseline characteristics with the differences were subsequently refined using LASSO regression analysis to identify 9 predictive indicators. Age, industry, mental health, marital status, general health status, education level, family income-to-poverty ratio, behavior, and anxiety were included in the most streamlined and regularized models, which the cross-validated error fell within 1 standard error of the minimum (optimal lambda = 0.00052). Figure 2A and B illustrates the coefficient distribution of the LASSO regression model and the cross-validation error plot.

### 3.3. Establishment and verification of a nomogram of predictive indicators in concussion

**Figure 3.**
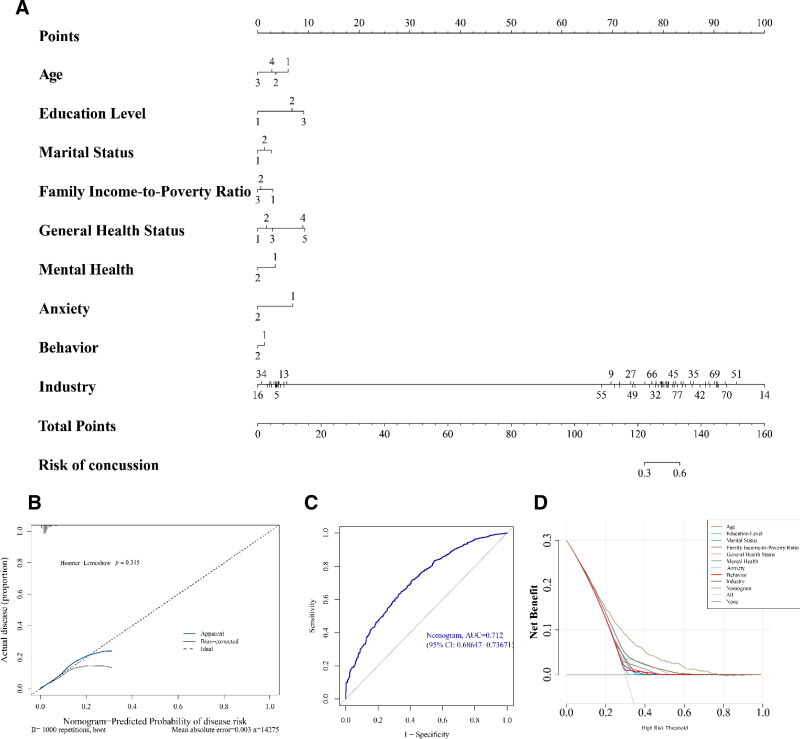
(A) Nomogram of predictive indicators in concussion. In the prediction model, each variable represents a risk factor. The line segments corresponding to each variable are marked with scales, indicating the range of possible values for that variable. The length of the line segment reflects the contribution of that factor to the outcome event. The line graph on the line segment illustrates the distribution of gene expression levels in the dataset. The single-item score (points) in the figure indicates the individual score for each variable at different values. The total score (total points) in the figure represents the cumulative total score from all variables when their values are applied; (B) The calibration curves to assess the predictive ability of the nomogram model; (C) ROC curve for evaluating the diagnostic worth of the nomogram; (D) DCA for evaluating the diagnostic worth of the nomogram. DCA = decision curve analysis, ROC = receiver operating characteristic

To construct a more practical and stable nomogram, predictive indicators were integrated to quantify the predicted survival probabilities for individuals. The higher the total points, the greater the likelihood of patient survival. The nomogram indicated that these factors significantly predict outcomes for patients with concussion, with higher points associated with a higher likelihood of concussion (Fig. 3A). The nomogram’s calibration curves demonstrated a strong concordance between predicted probability of survival and reference line. The H–L test (*P* = .315) suggested no notable disparity between the observed and predicted values, and the mean absolute error = 0.003 indicated a very small discrepancy between the predicted and actual risk, highlighting the high accuracy of the nomogram model in predicting concussion (Fig. 3B). A subsequent ROC curve analysis validated the nomogram’s predictive value, with AUC values of 0.712, signifying that the nomogram model had high diagnostic value (Fig. 3C). To further ascertain the nomogram’s clinical decision-making value, DCA was conducted. It was found that the nomogram outperformed individual variables in predicting concussion (Fig. 3D).

## 4. Discussion

Concussion, as a form of mild TBI, represents a significant yet frequently overlooked public health concern, primarily due to its nonspecific and transient symptoms, which pose diagnostic challenges.^[24]^ This study leveraged data from the 2023 NHIS to develop and validate a concussion risk prediction model. The model incorporates 9 key brain injury related factors: age, education level, general health status, family income-to-poverty ratio, marital status, mental health, anxiety, behavior, and industry. The resulting nomogram offers an innovative tool for clinical decision-making and patient management, enabling the identification of high-risk individuals, facilitating personalized treatment strategies, and providing a foundation for future research.

This study comprehensively considered the impact of demographic characteristics, socioeconomic status, and mental health conditions on the risk of concussion, and these dimensions were accordingly incorporated into the selection criteria for model development. A growing body of literature has established that age, socioeconomic disadvantage, and mental health disorders constitute major risk factors for concussion incidence.^[25,26]^ Young adults aged 18 to 44 years are at increased risk due to greater involvement in high-risk activities such as competitive sports and physically demanding occupations.^[27,28]^ In contrast, older adults (≥65 years) tend to experience more severe outcomes, including a heightened susceptibility to neurodegenerative sequelae including chronic traumatic encephalopathy and Alzheimer disease, owing to age-related vulnerability and diminished neural plasticity.^[7–9,29,30]^ These findings are consistent with the observations in our dataset, wherein the long-term consequences of concussion appeared more pronounced among older populations, reinforcing the notion that age moderates both the incidence and recovery trajectory of concussion.

In addition, socioeconomic determinants were found to exert a significant influence on concussion occurrence and prognosis. Individuals who were unmarried or divorced, had lower educational attainment, or reported annual incomes below $35,000 were observed to be at elevated risk of concussion, a pattern corroborated by previous epidemiological evidence.^[26,31–34]^ Our results further confirmed that patients from socioeconomically disadvantaged backgrounds may experience poorer recovery outcomes following concussion, especially among those with limited educational or financial resources, possibly due to reduced access to healthcare, limited social support, or delayed intervention.

Psychological factors, particularly anxiety, were also strongly associated with concussion risk. Prior studies have demonstrated that individuals with preexisting anxiety disorders are more likely to develop persistent postconcussive symptoms, such as emotional dysregulation, cognitive disturbances, and somatic complaints.^[35–37]^ These associations were similarly evident in our analysis, thereby supporting the hypothesized bidirectional relationship between concussion and psychological morbidity.^[25,38–41]^ This highlights the importance of incorporating mental health screening into both the prevention and post-injury care protocols for individuals at risk.

Moreover, occupational exposure was a salient factor in concussion susceptibility. Our findings indicated that individuals employed in performance arts, spectator sports, or occupations with recurrent head impact exposure were disproportionately represented among those reporting concussion.^[42–44]^ This elevated risk may stem from routine engagement in vigorous physical activity, contact sports, or high-impact environments that increase the likelihood of head trauma. Such occupational subgroups warrant heightened surveillance and targeted preventive strategies – including education, protective equipment, and workplace policy modifications – to mitigate concussion risk.^[41,44,45]^

Collectively, these findings not only align closely with the current body of literature concerning the role of age, socioeconomic conditions, psychological factors, and occupational context in concussion vulnerability, but also reinforce their cumulative relevance in guiding evidence-based prevention and intervention efforts. The present study thus provides a robust empirical basis for tailoring concussion risk mitigation strategies to high-risk populations identified by these multidimensional predictors.

Lifestyle factors, including alcohol consumption, drug use, and behavioral health, significantly influence concussion incidence and recovery.^[46,47]^ Individuals on long-term medications for mood, attention, or behavioral issues may face increased concussion risk due to pharmacological effects on the brain. Our findings suggest that lifestyle and psychological-behavioral factors play critical roles in concussion occurrence and recovery, necessitating a comprehensive evaluation of patients’ overall health in clinical practice.

The concussion risk prediction model developed in this study, based on LASSO regression, offers multiple advantages. By integrating predictors such as sociodemographic, health, comorbidity, and lifestyle factors into a nomogram, it provides a comprehensive, personalized tool with high specificity and accuracy (AUC = 0.712), enabling clinicians to identify high-risk patients early for close monitoring and tailored management.^[48–50]^ Existing brain injury prediction models mainly focus on prognostic evaluation after injury and rely on clinical measurement indicators. For example, a study published in Neurology in 2024 used prehospital lactate levels combined with the glasgow coma scale score to predict mortality in TBI patients, achieving an AUC of 0.874^[51]^; another 2022 study in Lancet Neurology used 2 biomarkers, glial fibrillary acidic protein and Ubiquitin C-terminal hydrolase L1 (UCHL1), to predict the risk of death or severe disability 6 months after TBI, with an accuracy of 87% to 89%.^[52]^ Although these models have high predictive accuracy, they are aimed at clinical decision-making after injury and cannot perform risk screening before onset. In contrast, the model in this study is the first to systematically integrate nonclinical, easily obtainable factors such as socioeconomic status (e.g., the ratio of household income to the poverty line) and mental health (e.g., anxiety levels), filling the gap in the process of “early risk identification – population stratification prevention.” Even compared with other disease prediction models that also employ LASSO regression, all 9 indicators included in this study are derived from routine health surveys and require no additional laboratory tests or imaging, thus making the model more suitable for large-scale application in settings such as primary healthcare institutions, community health service centers, and occupational health management. As a cross-sectional study, we primarily focused on identifying key predictive factors and developing a preliminary model. Despite the lack of external validation, the current model retains significant value. Compared to traditional prognostic models, LASSO regression minimizes overfitting through variable selection, with all predictors grounded in robust clinical and epidemiological evidence and model stability validated via 1000 bootstrap resamplings. Calibration curves and a low mean absolute error (MAE = 0.003) demonstrate strong agreement between predicted and actual outcomes, while DCA confirms the nomogram’s superiority over single-variable predictions. This nomogram supports risk stratification based on patient characteristics, facilitating timely interventions to reduce long-term concussion sequelae and significantly enhancing patient management.^[53]^ At the same time, this model demonstrates practical value across multiple scenarios in concussion management. In emergency or acute care settings, it can rapidly assess the concussion risk level of trauma patients. Particularly for primary healthcare facilities with limited medical resources, the model can serve as a preliminary screening tool to reduce unnecessary neuroimaging examinations. Furthermore, for high-risk occupational groups of different age ranges (such as athletes) and older adults, the model enables regular risk assessment and can be integrated with sleep monitoring and psychological screening to establish a “risk warning–early intervention” cycle. Misdiagnosis or missed diagnosis of concussion often leads to repeated visits and examinations, increasing healthcare costs, while severe cases that progress to traumatic encephalopathy require long-term neurorehabilitation, further exacerbating the economic burden.^[54]^ Based on health economic data from similar predictive models, the standardized risk assessment process proposed in this study could help reduce unnecessary computed tomography examinations and alleviate financial strain.

Several limitations inherent to the present study warrant critical consideration. Foremost, the predictive model was developed and internally validated exclusively using the 2023 NHIS dataset, thus lacking external validation. This constrains the generalizability of the model to broader populations. Future investigations should aim to incorporate independent datasets – such as earlier NHIS waves or alternative nationally representative health databases – to rigorously assess the model’s external validity across diverse cohorts. Second, the cross-sectional design of this study inherently precludes any inference regarding temporal associations or long-term trajectories. As such, the model is unable to capture dynamic changes in concussion outcomes over time – such as the progression toward cognitive decline or the development of chronic traumatic encephalopathy. Longitudinal, prospective studies are essential to elucidate the temporal stability and predictive robustness of the proposed model across various stages of concussion recovery. Third, the reliance on self-reported questionnaire data for the identification of concussion cases introduces the potential for recall bias and misclassification, particularly in the absence of confirmatory clinical evaluation or biomarker-based diagnostics (e.g., neuroimaging findings or tau protein quantification). Additionally, class imbalance within the analytic dataset may have influenced model calibration. To enhance diagnostic precision and model performance, future research should consider expanding the sample size and incorporating multimodal data sources – including clinical, imaging, and molecular biomarker data. Despite these limitations, the present study establishes a foundational framework for the development of concussion risk stratification tools. Addressing the aforementioned limitations through robust external validation, longitudinal data integration, and the inclusion of objective diagnostic modalities will be critical to advancing the model’s translational potential and guiding clinical implementation in personalized concussion management paradigms.

## 5. Conclusion

This study successfully developed and validated a concussion risk prediction model based on 2023 NHIS data. By identifying key brain injury related factors, the model offers clinicians a promising tool to detect high-risk individuals and deliver personalized interventions as needed. Despite its robust predictive performance and clinical relevance, further validation through external datasets, longitudinal studies and the inclusion of additional biomarkers is necessary to enhance its accuracy and applicability. As the burden of concussions grows, the development of such predictive tools will be pivotal in early detection, management, and improving patient outcomes.

## Author contributions

**Conceptualization:** Senyuan Yang, Yashi Chen, Shunqiu Huang, Yeling Deng.

**Data curation:** Senyuan Yang, Yashi Chen, Shunqiu Huang.

**Formal analysis:** Senyuan Yang, Yashi Chen, Shunqiu Huang.

**Investigation:** Senyuan Yang.

**Methodology:** Yashi Chen.

**Writing – original draft:** Yeling Deng, Xiaobin Zhou, Yong Li.

**Writing – review & editing:** Senyuan Yang, Yashi Chen, Shunqiu Huang, Xiaobin Zhou, Yong Li.

## Supplementary Material



## References

[1] Haarbauer-Krupa J Pugh MJ Prager EM Harmon N Wolfe J Yaffe K. Epidemiology of chronic effects of traumatic brain injury. J Neurotrauma. 2021;38:3235–47. 33947273 10.1089/neu.2021.0062 PMC9122127

[2] Maas AIR Menon DK Adelson PD; InTBIR Participants and Investigators. Traumatic brain injury: integrated approaches to improve prevention, clinical care, and research. Lancet Neurol. 2017;16:987–1048. 29122524 10.1016/S1474-4422(17)30371-X

[3] Harmon KG Drezner JA Gammons M. American medical society for sports medicine position statement: concussion in sport. Br J Sports Med. 2013;47:15–26. 23243113 10.1136/bjsports-2012-091941

[4] Willer B Leddy JJ. Management of concussion and post-concussion syndrome. Curr Treat Options Neurol. 2006;8:415–26. 16901381 10.1007/s11940-006-0031-9

[5] Leddy JJ Haider MN Noble JM. Clinical assessment of concussion and persistent post-concussive symptoms for neurologists. Curr Neurol Neurosci Rep. 2021;21:70. 34817724 10.1007/s11910-021-01159-2

[6] Voormolen DC Polinder S von Steinbuechel N Vos PE Cnossen MC Haagsma JA. The association between post-concussion symptoms and health-related quality of life in patients with mild traumatic brain injury. Injury. 2019;50:1068–74. 30554897 10.1016/j.injury.2018.12.002

[7] Buckland ME Affleck AJ Pearce AJ Suter CM. Chronic traumatic encephalopathy as a preventable environmental disease. Front Neurol. 2022;13:880905. 35769361 10.3389/fneur.2022.880905 PMC9234108

[8] Brett BL Gardner RC Godbout J Dams-O’Connor K Keene CD. Traumatic brain injury and risk of neurodegenerative disorder. Biol Psychiatry. 2022;91:498–507. 34364650 10.1016/j.biopsych.2021.05.025 PMC8636548

[9] Bieniek KF Blessing MM Heckman MG. Association between contact sports participation and chronic traumatic encephalopathy: a retrospective cohort study. Brain Pathol. 2020;30:63–74. 31199537 10.1111/bpa.12757 PMC6916416

[10] Kerr ZY Roos KG Djoko A. Epidemiologic measures for quantifying the incidence of concussion in national collegiate athletic association sports. J Athl Train. 2017;52:167–74. 27331336 10.4085/1062-6050-51.6.05 PMC5384815

[11] Veliz P Eckner JT Zdroik J Schulenberg JE. Lifetime prevalence of self-reported concussion among adolescents involved in competitive sports: a national U.S. study. J Adolesc Health. 2019;64:272–5. 30409755 10.1016/j.jadohealth.2018.08.023 PMC6339843

[12] Karr JE Areshenkoff CN Duggan EC Garcia-Barrera MA. Blast-related mild traumatic brain injury: a Bayesian random-effects meta-analysis on the cognitive outcomes of concussion among military personnel. Neuropsychol Rev. 2014;24:428–44. 25253505 10.1007/s11065-014-9271-8

[13] Rosenfeld JV McFarlane AC Bragge P Armonda RA Grimes JB Ling GS. Blast-related traumatic brain injury. Lancet Neurol. 2013;12:882–93. 23884075 10.1016/S1474-4422(13)70161-3

[14] Martha SR Tolentino EJ Bugajski AA Thompson HJ. Telomere length associates with symptom severity after mild traumatic brain injury in older adults. Neurotrauma Rep. 2023;4:350–8. 37284700 10.1089/neur.2023.0012 PMC10240314

[15] Hadanny A Efrati S. Treatment of persistent post-concussion syndrome due to mild traumatic brain injury: current status and future directions. Expert Rev Neurother. 2016;16:875–87. 27337294 10.1080/14737175.2016.1205487

[16] Riegler KE Echemendia R Meeuwisse W. Examining the reliability and validity of coding perceived force severity and bracing in the NHL concussion spotter program. Orthop J Sports Med. 2024;12:23259671241285075. 39534394 10.1177/23259671241285075 PMC11555722

[17] Castellanos J Phoo CP Eckner JT; CARE Consortium Investigators. Predicting risk of sport-related concussion in collegiate athletes and military cadets: a machine learning approach using baseline data from the CARE consortium study. Sports Med. 2021;51:567–79. 33368027 10.1007/s40279-020-01390-w

[18] Ramulu PK Belagaje SR Varadaraj V. Association of concussion/brain injury symptoms and diagnosis with mental and social well-being in 2020 National Health Interview Survey (NHIS) children. Brain Inj. 2024;38:620–9. 38664868 10.1080/02699052.2024.2328312

[19] Panos A Mavridis D. TableOne: an online web application and R package for summarising and visualising data. Evid Based Ment Health. 2020;23:127–30. 32665250 10.1136/ebmental-2020-300162 PMC10231609

[20] Friedman J Hastie T Tibshirani R. Regularization paths for generalized linear models via coordinate descent. J Stat Softw. 2010;33:1–22. 20808728 PMC2929880

[21] Sachs MC. plotROC: a tool for plotting ROC curves. J Stat Softw. 2017;79:2. 30686944 10.18637/jss.v079.c02 PMC6347406

[22] Zhang Z Rousson V Lee WC; written on behalf of AME Big-Data Clinical Trial Collaborative Group. Decision curve analysis: a technical note. Ann Transl Med. 2018;6:308. 30211196 10.21037/atm.2018.07.02 PMC6123195

[23] Robin X Turck N Hainard A. pROC: an open-source package for R and S+ to analyze and compare ROC curves. BMC Bioinf. 2011;12:77. Published 2011 Mar 17 10.1186/1471-2105-12-77 PMC3068975 21414208

[24] Master CL Mayer AR Quinn D Grady MF. Concussion. Ann Intern Med. 2018;169:ITC1–ITC16. 29971425 10.7326/AITC201807030

[25] Memmini AK Mosesso KM Perkins SM; CARE Consortium Investigators. Premorbid risk factors and acute injury characteristics of sport-related concussion across the national collegiate athletic association: findings from the concussion assessment, research, and education (CARE) consortium. Sports Med. 2023;53:1457–70. 36929588 10.1007/s40279-023-01830-3

[26] Cook NE Gaudet CE Iverson GL. Association between social determinants of health and concussion among high school students in the United States. J Child Neurol. 2025;40:278–90. 39819208 10.1177/08830738241304867

[27] McCrory P Meeuwisse W Dvořák J. Consensus statement on concussion in sport-the 5th international conference on concussion in sport held in Berlin, October 2016. Br J Sports Med. 2017;51:838–47. 28446457 10.1136/bjsports-2017-097699

[28] Tsushima WT Siu AM Ahn HJ Chang BL Murata NM. Incidence and risk of concussions in youth athletes: comparisons of age, sex, concussion history, sport, and football position. Arch Clin Neuropsychol. 2019;34:60–9. 29554189 10.1093/arclin/acy019 PMC6345352

[29] Morissette MP Prior HJ Tate RB Wade J Leiter JRS. Associations between concussion and risk of diagnosis of psychological and neurological disorders: a retrospective population-based cohort study. Fam Med Community Health. 2020;8:e000390. 32719017 10.1136/fmch-2020-000390 PMC7388873

[30] Radhakrishnan R Garakani A Gross LS. Neuropsychiatric aspects of concussion. Lancet Psychiatry. 2016;3:1166–75. 27889010 10.1016/S2215-0366(16)30266-8

[31] Mascialino G Perrin PB Arango-Lasprilla JC Watson JD Rodríguez-Lorenzana A Paz C. Marital stability during the year after traumatic brain injury in an ecuadorian sample: a repeated-measures study. J Clin Med. 2024;13:7169. 39685628 10.3390/jcm13237169 PMC11642071

[32] Boltz AJ Memmini AK Brett BL; CARE CONSORTIUM INVESTIGATORS†. Intersection of race and socioeconomic status on concussion recovery among NCAA student-athletes: a CARE consortium study. Med Sci Sports Exerc. 2023;55:2180–93. 37486776 10.1249/MSS.0000000000003258

[33] Hunt TN Roberts K Taylor EM Quintana CP Kossman MK. The effect of social determinants of health on clinical recovery following concussion: a systematic review. J Sport Rehabil. 2024;34:28–36. Published 2024 Mar 20 38508176 10.1123/jsr.2023-0068

[34] Mikolić A van Klaveren D Jost M; CENTER-TBI Participants and Investigators. Prognostic models for depression and post-traumatic stress disorder symptoms following traumatic brain injury: a CENTER-TBI study. BMJ Ment Health. 2025;28:e301181. Published 2025 Jan 15 10.1136/bmjment-2024-301181 PMC11751936 39819833

[35] McCarty CA Zatzick DF Marcynyszyn LA. Effect of collaborative care on persistent postconcussive symptoms in adolescents: a randomized clinical trial. JAMA Netw Open. 2021;4:e210207. Published 2021 Feb 1 33635325 10.1001/jamanetworkopen.2021.0207 PMC7910815

[36] Chrisman SPD Whitlock KB Mendoza JA. Pilot randomized controlled trial of an exercise program requiring minimal in-person visits for youth with persistent sport-related concussion [published correction appears in Front Neurol. 2020 Feb 21;11:6. doi: 10.3389/fneur.2020.00006.]. Front Neurol. 2019;10:623. Published 2019 Jun 17 31316446 10.3389/fneur.2019.00623 PMC6611408

[37] McCarty CA Zatzick D Stein E Wang J Hilt R Rivara FP; Seattle Sports Concussion Research Collaborative. Collaborative care for adolescents with persistent postconcussive symptoms: a randomized trial. Pediatrics. 2016;138:e20160459. 27624513 10.1542/peds.2016-0459 PMC5051206

[38] Joyce JM Mercier LJ Stokoe M. Glutamate, GABA and glutathione in adults with persistent post-concussive symptoms. Neuroimage Clin. 2022;36:103152. 36007438 10.1016/j.nicl.2022.103152 PMC9424629

[39] Fish AM Vanni J Mohammed FN. Comparison of anxiety and depression symptoms in concussed and nonconcussed adolescents. Sports Health. 2023;15:185–91. 35919017 10.1177/19417381221113840 PMC9950990

[40] Patricios JS Schneider KJ Dvorak J. Consensus statement on concussion in sport: the 6th international conference on concussion in sport-amsterdam, October 2022. Br J Sports Med. 2023;57:695–711. 37316210 10.1136/bjsports-2023-106898

[41] Manley G Gardner AJ Schneider KJ. A systematic review of potential long-term effects of sport-related concussion. Br J Sports Med. 2017;51:969–77. 28455362 10.1136/bjsports-2017-097791 PMC5466926

[42] Gallo V McElvenny DM Seghezzo G. Concussion and long-term cognitive function among rugby players-The BRAIN Study. Alzheimers Dement. 2022;18:1164–76. 34668650 10.1002/alz.12455 PMC9298292

[43] Dahlén SC Bjørneboe J Sandmo SK. Brain health in Norwegian female former top-level football players: a protocol for a longitudinal cohort study. BMJ Open. 2025;15:e092456. Published 2025 Jan 11 10.1136/bmjopen-2024-092456 PMC11751818 39800397

[44] Chandran A Boltz AJ Baker J Anderson M Rao N Collins CL. Concussion in high school sports: findings from injury surveillance. Pediatr Res. 2025;98:1718–25. 39833348 10.1038/s41390-025-03863-y

[45] Yeates KO Räisänen AM Premji Z. What tests and measures accurately diagnose persisting post-concussive symptoms in children, adolescents and adults following sport-related concussion? A systematic review. Br J Sports Med. 2023;57:780–8. 37316186 10.1136/bjsports-2022-106657

[46] Knell G Burkhart SO Caze TJ Polousky JD Kohl HW 3rd Messiah SE. Association between concussion history and factors relating to cognitive, behavioral, and emotional health among american high school athletes: a cross-sectional analysis. Am J Sports Med. 2020;48:2534–43. 32692937 10.1177/0363546520938776

[47] Benoy R Ramirez C Hitchcock M Reardon C. Cannabis use in adolescent and young adult athletes: a clinical review. Sports Health. 2024;16:279–84. 37950433 10.1177/19417381231208661 PMC10916776

[48] Li X Cai D Mei C Huang X. Construction and validation of a predictive model for mortality risk in patients with acinetobacter baumannii bloodstream infection. Infect Drug Resist. 2024;17:5247–60. Published 2024 Nov 26 39624638 10.2147/IDR.S491537 PMC11609411

[49] Xu J Wang X Chen W Tian M You C. Incorporating platelet-to-white blood cell ratio into survival prediction models for intracerebral hemorrhage: a nomogram approach. Front Neurol. 2024;15:1464216. Published 2024 Oct 10 39450047 10.3389/fneur.2024.1464216 PMC11499137

[50] Zou K Huang S Ren W. Development and validation of a dynamic nomogram for predicting in-hospital mortality in patients with acute pancreatitis: a retrospective cohort study in the intensive care unit. Int J Gen Med. 2023;16:2541–53. Published 2023 Jun 17 37351008 10.2147/IJGM.S409812 PMC10284301

[51] Martin-Rodriguez F Sanz-Garcia A Lopez-Izquierdo R. Prehospital lactate levels obtained in the ambulance and prediction of 2-day in-hospital mortality in patients with traumatic brain injury. Neurology. 2024;103:e209692. 39088773 10.1212/WNL.0000000000209692

[52] Korley FK Jain S Sun X; TRACK-TBI Study Investigators. Prognostic value of day-of-injury plasma GFAP and UCH-L1 concentrations for predicting functional recovery after traumatic brain injury in patients from the US TRACK-TBI cohort: an observational cohort study. Lancet Neurol. 2022;21:803–13. 35963263 10.1016/S1474-4422(22)00256-3 PMC9462598

[53] Peng J Chen J Yin C Zhang P Yang J. Comparison of machine learning models in predicting mental health sequelae following concussion in youth. AMIA Jt Summits Transl Sci Proc. 2025;2025:422–31. 40502265 PMC12150734

[54] Kontos AP Sufrinko A Sandel N Emami K Collins MW. Sport-related concussion clinical profiles: clinical characteristics, targeted treatments, and preliminary evidence. Curr Sports Med Rep. 2019;18:82–92. 30855306 10.1249/JSR.0000000000000573

