# Supplementary material for: Development and validation of a concussion risk prediction model using 2023 National Health Interview Survey (NHIS) data
Source: Medicine (Baltimore). 2026 Mar 6;105(10):e47935. doi: 10.1097/MD.0000000000047935 (PMC12975239; doi:10.1097/MD.0000000000047935)
Supplement: Supplementary file 1 [file medi-105-e47935-s001.docx]

**Supplementary Table 1**

| **Variable** | **Description** | **Answer Options** |
| --- | --- | --- |
| TBILCDCMG_A | During the past 12 months, as a result of a blow or jolt to the head, have you been knocked out or lost consciousness, been dazed or confused, or had a gap in your memory? | Yes/No |
| TBIHLSBMC_A | During the past 12 months, as a result of a blow or jolt to the head, have you had headaches, sensitivity to light or noise, balance problems, or changes in mood or behavior? | Yes/No |
| TBISPORT_A | Were you playing a sport or participating in a physical or recreational activity, such as jogging, biking, or pick-up games, when you experienced any of these blows or jolts to the head? | Yes/No |
| TBILEAGUE_A | Were you participating in an organized team or league sports competition or practice when you experienced any of these blows or jolts to the head? | Yes/No |
| TBIEVAL_A | During the past 12 months, as a result of a blow or jolt to the head, were you evaluated for a concussion or brain injury by a doctor, nurse, paramedic, athletic trainer, or other health professional? | Yes/No |

**Supplementary Table 1**

This table lists the five questionnaires used to identify concussions, all of which are derived from the 2023 National Health Interview Survey (NHIS) data. These questionnaires assess whether individuals experienced a blow or jolt to the head in the past 12 months, along with related symptoms and whether they received a concussion evaluation. Individuals who answered "Yes" to these questionnaires are classified as having a concussion.

**Supplementary Table 2**

| No. | Variable | Dataset and Definition Code | Variable Meaning | Year |
| --- | --- | --- | --- | --- |
| 1 | Age | AGEP_A | 18-24; 25-44; 45-64; ≥65 | 2023 |
| 2 | Gender | SEX_A | Male; Female | 2023 |
| 3 | Race | HISDETP_A | Hispanic (Mexican/Mexican American); Hispanic (all other groups); Non-Hispanic | 2023 |
| 4 | Education Level | EDUCP_A | High school or below; High school; High school or above | 2023 |
| 5 | Marital Status | MARITAL_A | Married; Unmarried cohabitating; Neither | 2023 |
| 6 | Family Income-to-Poverty Ratio | RATCAT_A | <35,000 USD; 35,000-64,999 USD; ≥65,000 USD | 2023 |
| 7 | BMI | BMICAT_A | Underweight; Healthy weight; Overweight; Obesity | 2023 |
| 8 | General Health Status | PHSTAT_A | Excellent; Very good; Good; Fair; Poor | 2023 |
| 9 | Mental Health | MHTHRPY_A | Yes; No | 2023 |
| 10 | Health Insurance | NOTCOV_A | Not purchased; Purchased | 2023 |
| 11 | Anxiety | ANXEV_A | Yes; No | 2023 |
| 12 | Behavior | MHRX_A | Yes; No | 2023 |
| 13 | Industry | EMDINDSTN1_A | Crop production; Animal production and aquaculture; Forestry and logging; Fishing, hunting, and trapping; Support activities for agriculture and forestry; Oil and gas extraction; Mining (except oil and gas); Support activities for mining; Utilities; Construction; 1Food manufacturing; 1Beverage and tobacco product manufacturing; 1Textile mills; 1Textile product mills; 1Apparel manufacturing; 1Leather and allied product manufacturing; 1Wood products manufacturing; 1Paper manufacturing; 1Printing and related support activities; Petroleum and coal products manufacturing; Chemical manufacturing; Plastics and rubber products manufacturing; Nonmetallic mineral product manufacturing; Primary metal manufacturing; Fabricated metal product manufacturing; Machinery manufacturing; Computer and electronic product manufacturing; Electrical equipment, appliances, and components manufacturing; Transportation equipment manufacturing; Furniture and related products manufacturing; Miscellaneous manufacturing; Wholesale trade, durable goods; Wholesale trade, nondurable goods; Nondesignated wholesale trade; Motor vehicle and parts dealers; Furniture and home furnishings stores; Electronics and appliance stores; Building material and garden equipment and supplies dealers; Food and beverage stores; Health and personal care stores; Gas stations; Clothing, footwear, jewelry, luggage, and leather goods stores; Sporting goods, hobby, book, and music stores; Department stores; 4Miscellaneous store retailers; Non-store retailers and non-designated retail trade; Transportation (including transportation support activities); Postal services, couriers, and messengers; Warehousing and storage; Newspaper, periodical, book, and software publishing; Motion picture and sound recording industries; Broadcasting and telecommunications; Libraries and archives, internet publishing, web search portals, data processing and hosting services, and other information services; Monetary authorities - central bank; Credit intermediaries and related activities; Securities, commodity contracts, and other financial investments and related activities; Insurance carriers and related activities; Real estate; Automotive and other consumer goods rental services; Commercial, industrial, and other intangible asset services (excluding copyrighted works); Professional, scientific, and technical services; Management of companies and enterprises; Administrative and support and waste management and remediation services; Educational services; Ambulatory health care services; Hospitals; Nursing and residential care facilities; Social assistance; Performing arts, spectator sports, and related industries; Museums, historical sites, and similar institutions; Amusement, gambling, and recreation industries; Accommodations; Food services and drinking places; Repair and maintenance; Personal services (e.g., barbershops, beauty salons, nail salons, laundromats, funeral homes, and cemeteries); Religious organizations, grantmaking organizations, civic organizations, labor organizations, professional organizations, and similar organizations; Private households; Public administration; Armed forces | 2023 |
| 14 | Occupation | EMDOCCUPN2_A | Management occupations; Business and financial operations occupations; Computer and mathematical occupations; Architecture and engineering occupations; Life, physical, and social science occupations; Community and social service occupations; Legal occupations; Education, training, and library occupations; Arts, design, entertainment, sports, and media occupations; Healthcare practitioners and technical occupations; 1Healthcare support occupations; Protective service occupations; Food preparation and serving-related occupations; Building and grounds cleaning and maintenance occupations; Personal care and service occupations; Sales and related occupations; Office and administrative support occupations; Farming, fishing, and forestry occupations; Construction and extraction occupations; Installation, maintenance, and repair occupations; Production occupations; Transportation and material moving occupations; Military-specific occupations | 2023 |

**Supplementary Table 2**

This table presents a list of covariates included in the study to account for potential confounding effects on concussion risk. The variables are categorized into demographic characteristics, health status, comorbidities, lifestyle factors, and occupational data
